# Mechanochemically Induced Solid-State Transformations of Levofloxacin

**DOI:** 10.1021/acs.molpharmaceut.4c00008

**Published:** 2024-04-25

**Authors:** Lena Kadri, Maria Carta, Giulio Lampronti, Francesco Delogu, Lidia Tajber

**Affiliations:** † School of Pharmacy and Pharmaceutical Sciences, Trinity College Dublin, College Green, Dublin 2 D02 PN40, Ireland; ‡ The Science Foundation Ireland Research Centre for Pharmaceuticals (SSPC), Limerick V94 T9PX, Ireland; § Department of Mechanical, Chemical and Materials Engineering, University of Cagliari, CSGI Research Unit, via Marengo 2, Cagliari 09123, Italy; ∥ Department of Materials Science & Metallurgy, University of Cambridge, 27 Charles Babbage Road, Cambridge CB3 0FS, United Kingdom

**Keywords:** levofloxacin, fluoroquinolone, mechanochemistry, ball milling, spray drying, phase transformation, kinetics

## Abstract

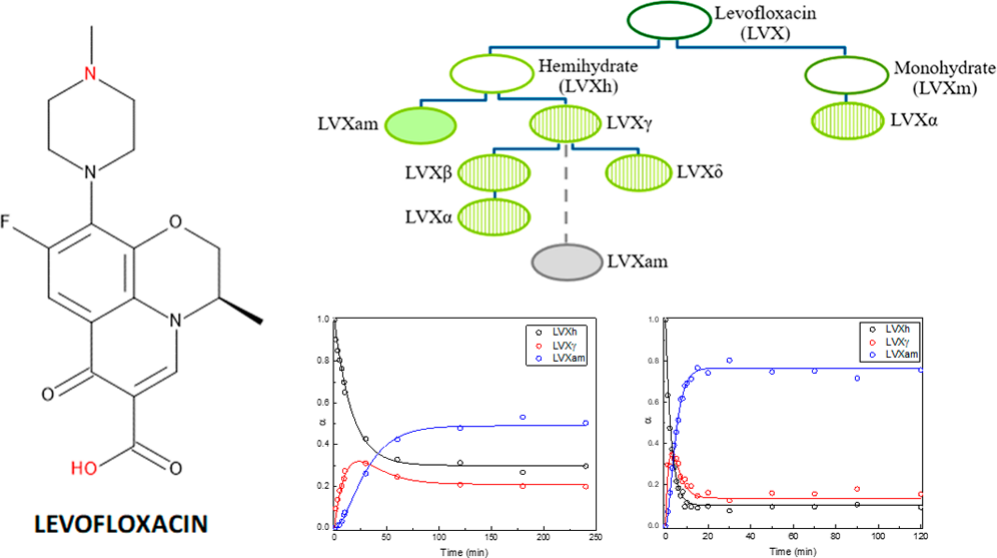

Levofloxacin hemihydrate (LVXh) is a complex fluoroquinolone drug that exists in both hydrated and anhydrous/dehydrated forms. Due to the complexity of such a compound, the primary aim of this study was to investigate the amorphization capabilities and solid-state transformations of LVXh when exposed to mechanical treatment using ball milling. Spray drying was utilized as a comparative method for investigating the capabilities of complete LVX amorphous (LVXam) formation. The solid states of the samples produced were comprehensively characterized by powder X-ray diffraction, thermal analysis, infrared spectroscopy, Rietveld method, and dynamic vapor sorption. The kinetics of the process and the quantification of phases at different time points were conducted by Rietveld refinement. The impact of the different mills, milling conditions, and parameters on the composition of the resulting powders was examined. A kinetic investigation of samples produced using both mills disclosed that it was in fact possible to partially amorphize LVXh upon mechanical treatment. It was discovered that LVXh first transformed to the anhydrous/dehydrated form γ (LVXγ), as an intermediate phase, before converting to LVXam. The mechanism of LVXam formation by ball milling was successfully revealed, and a new method of forming LVXγ and LVXam by mechanical forces was developed. Spray drying from water depicted that complete amorphization of LVXh was possible. The amorphous form of LVX had a glass transition temperature of 80 °C. The comparison of methods highlighted that the formation of LVXam is thus both mechanism- and process-dependent. Dynamic vapor sorption studies of both LVXam samples showed comparable stability properties and crystallized to the most stable hemihydrate form upon analysis. In summary, this work contributed to the detailed understanding of solid-state transformations of essential fluoroquinolones while employing greener and more sustainable manufacturing methods.

## 1 Introduction

Mechanochemistry is the area of science that addresses the chemical reactions initiated and driven by the application of mechanical forces to chemical systems involving at least one solid phase.^1−3^ Mechanochemistry has been utilized since the early 19th century.^4−7^ However, it was not until the 1960s that the processing of powders gained such traction, leading to further discoveries of mechanically activated solids resulting in amorphous and nanostructured phases in the 1980s.^1,8−12^ More recently, the well-known ability of mechanical forces to induce transformations in solids has attracted renewed attention from wide sectors of inorganic and organic synthetic chemistry, becoming the most rapidly growing area of chemical sciences.^13,14^ In particular, it is the unprecedented capacity of performing chemical reactions in the absence of a solvent phase that recognized mechanochemistry as one of the top ten chemical innovations sought to change the world.^15^ Accordingly, significant emphasis has been placed on the mechanochemical synthesis of fine chemicals and pharmaceuticals.^16−19^

Solid-state transformations of active pharmaceutical ingredients (APIs) have been reported by milling as a form of mechanical treatment. This was the case for sulfathiazole whereby polymorphism was detected as a direct result of mechanical activation by the process of milling.^20^ Sulfathiazole polymorphic forms I and III also demonstrated the capability to further transform to the amorphous phase upon further processing. Following recrystallization, depending on the polymorph, the return to either the pure polymorphic form or a mixture of polymorphs was plausible.^21^ Further examples of pharmaceutical materials exhibiting polymorphic transformations on mechanical treatment include chloramphenicol palmitate,^22^ mannitol,^23^ and sorbitol.^24^

Fluoroquinolone drugs are broad-spectrum antimicrobial agents, active against Gram-positive and negative bacteria.^25^ Many fluoroquinolone drugs are included on the World Health Organization (WHO) essential medicines list. For oral solid dosage forms, ciprofloxacin (CIP) is listed as a watch group antibiotic due to its high probability of developing bacterial resistance.^26^ Additionally, it is classified as one of the most critically important antimicrobials for human medicine.^27^ Moxifloxacin (MOX) and levofloxacin (LVX) are listed as essential antituberculosis medicines. For solutions, ofloxacin (OFL) is listed as part of the essential ophthalmological preparations for anti-infective agents. In addition, OFL and CIP are listed as essential ear, nose, and throat medicines.^26^

Research shows that multiple fluoroquinolones have been ball milled (BMed) to improve the properties of the drugs, such as solubility and dissolution rates, reduce particle size, enhance crystal engineering,^28^ and detect new polymorphic forms.^29^ Examples include CIP^30,31^ and enrofloxacin (ENRO),^32^ which were BMed with amino acids^9^ and acidic polymers^31,32^ to produce amorphous binary and ternary systems for solubility^30−32^ and dissolution enhancement.^32^ Norfloxacin (NOR), CIP, and ENRO were investigated via ball milling (BMing) to enhance the crystal engineering understanding of fluoroquinolone drugs. These fluoroquinolones were mixed with acid conformers and prepared by liquid-assisted grinding (LAG) to produce new molecular salts, ternary molecular ionic cocrystals, viscoelastic materials, and supramolecular gels.^28^ Besifloxacin was BMed to increase its low solubility in water and to improve the treatment of bacterial conjunctivitis. Nanocrystals were produced via wet bead milling to enhance dissolution, with the promising potential of improving bioavailability. Another investigation was carried out on gatifloxacin (GAT) whereby the amorphous form was achieved by milling and melt quenching to better understand the characteristics of various amorphous forms of GAT with multiple hydrate and anhydrous polymorphs being reported in their crystalline form.^29^ Also, CIP hydrochloride monohydrate was BMed to reduce its particle size before spray drying (SDing) to produce antibiotic inhalation powders.^33^

Spray drying (SDing) is another method that can be employed to alter the physicochemical properties of drugs. This method relies on the evaporative removal of solvent from droplets produced by atomization of a liquid feed comprising the API. The first drugs, which were processed by SDing, were phenylbutazone^34^ and thiazide diuretics.^35^ The antibiotic aztreonam was SDed without the use of an excipient to simplify the SDing process and to reduce side effects caused by excipient addition.^36^ However, excipients are known to improve the properties of micronized APIs, such as solubility,^37^ flowability,^38^ surface roughness, and particle dispersibility.^36^

**Figure 1:**
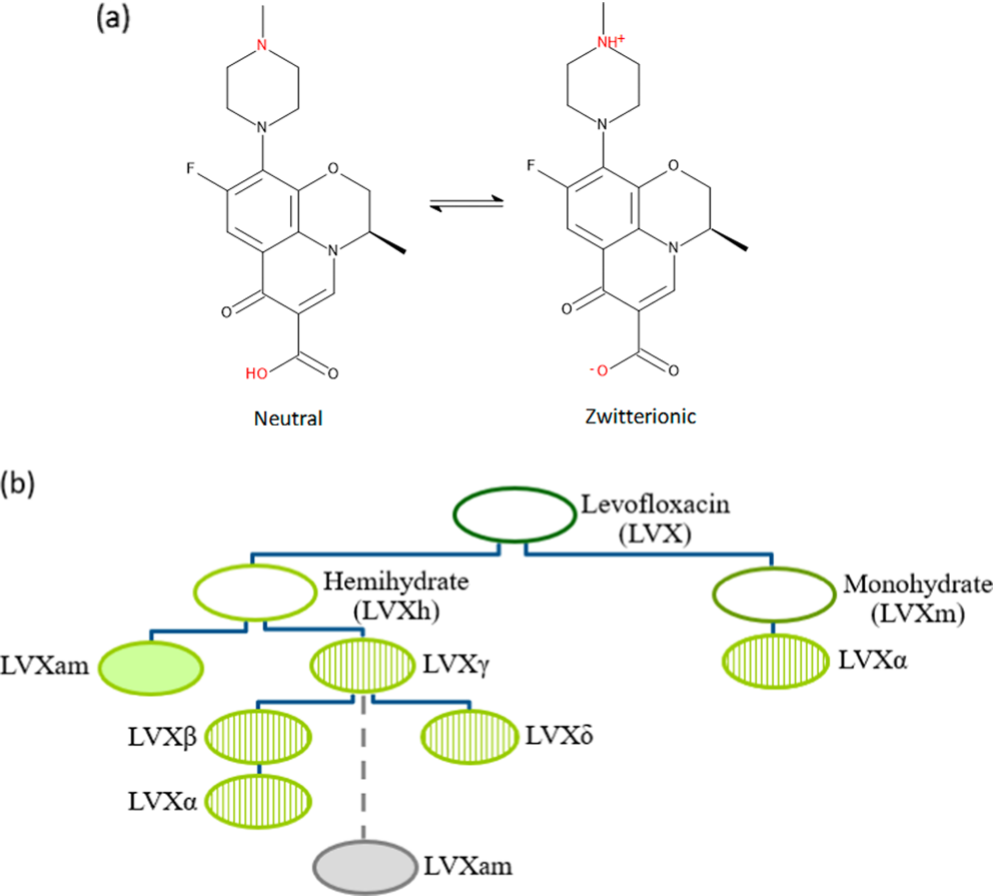
(a) Structural formula of LVX in its neutral and zwitterionic forms and (b) interconversion pathways of LVX to hydrates (open symbol), amorphous (filled symbol), and anhydrous/dehydrated forms (striped symbol).^39,42^ The broken line and gray symbol indicate a new pathway to amorphous LVX discovered in this work.

LVX was chosen for this research as it is a more complex fluoroquinolone drug in comparison to CIP and ENRO in terms of its solid-state forms. This complexity can be explained by the ability of LVX to exist in many different crystal forms.^39−43^ LVX can crystallize into two hydrate forms i.e., hemihydrate and monohydrate.^42^ Foremost, five water-free forms identified as amorphous, γ, δ, β, and α have been detected from the dehydration and heating of the known hydrate form LVXh in their solid phase.^39−43^ Moreover, levofloxacin monohydrate (LVXm) has only been reported to form anhydrous α following heated conditions in solution.^39−43^ Nevertheless, the crystal structure of the β and δ forms remain unidentified, while the structures of the hemihydrate, monohydrate, γ,^40^ and α^41^ forms are known.^40^ The interconversion pathways of the various forms of LVX can be observed in Figure 1.^39−43^

The first detection of the presence of anhydrous forms was reported after differential scanning calorimetry (DSC) analysis of LVXh whereby a broad endotherm between 20 and 70 °C indicated the loss of water, followed by the melting of anhydrous phases γ, β, and α at temperatures 225.4–234.1 °C.^39,42^ However, the further detection of the water-free crystal structures arose following either high-temperature powder X-ray diffraction (PXRD) analysis of anhydrous/dehydrated LVXγ (150 °C) from LVXh and anhydrous/dehydrated LVXα (190 °C) from LVXm^40^ or by desolvation of LVXh to form LVXγ and LVXα.^41^ The detection of partial amorphization from LVXh was hypothesized to be a result of dehydration and was only detected via DSC analysis.^42^ LVX is administered orally in its hemihydrate form (LVXh), due to its superior stability.^41^ The anhydrous/dehydrated forms associated with LVXh and LVXm are unstable at room temperature; thus, their presence may adversely impact the stability of LVX powder.

In this work, the utmost focus is on understanding the mechanisms behind solid-state transformations induced by a mechanochemical process as well as spray drying due to the possibility of solid conversions considering the ubiquity of these secondary pharmaceutical processes. The feasibility of obtaining an amorphous form of LVX was investigated by using both processes. However, a more in-depth investigation was performed for BMed samples to gain insights into the influence of BMing duration and the impact of utilizing different numbers of balls and varying the diameter of such balls. Thus, process variables were examined in the pursuit of obtaining amorphous LVX. The processed forms were characterized for the solid-state form by thermal analysis, and powder X-ray diffraction as well as the kinetics of the transformations were determined. The physical stability of the systems was established. This study is novel as LVX has never been BMed or SDed without the use of an excipient to successfully achieve amorphization. Furthermore, this account discusses the new discoveries made regarding the interconversion pathways of LVXh, LVXγ, and LVXam, as a result of milling LVXh rather than using the previous dehydration and desolvation methods for discovering solid-state forms.

## 2 Materials and Methods

### 2.1 Materials

LVX hemihydrate (LVXh) was obtained from Merck (U.K.). Deionized water (14.0 MΩ·cm) using an Elix 3 connected to a Synergy UV system (Millipore, U.K.) was used in all experiments. Potassium bromide Fourier transform infrared spectroscopy (FTIR) grade, ≥99% trace metal basis, was purchased from Merck (U.K.).

### 2.2 Methods

#### 2.2.1 Milling

##### 2.2.1.1 Retsch Planetary Ball Mill PM100

LVXh was BMed using a Retsch planetary ball mill PM100 (Germany) at 400 rpm. A 50 mL stainless steel grinding jar was used, and 2 g of sample was added to the jar. Three stainless steel milling balls with a diameter of 1.5 cm and a weight of 14 g each and eight balls with a diameter of 1.2 cm and a weight of 7 g were used for comparison purposes. Ball milling was conducted at room temperature for a maximum of 30 min at a time. A 15 min break was implemented after every 30 min of milling. The samples were milled for a total duration of 4 h at time point measurements taken every 1, 3, 5, 7, 9, and 10 min and again at 0.5, 1, 2, 3, and 4 h. The samples were taken by opening the jar and removing the sample amount required for analysis with a spatula. Once the sample was removed, the jar was closed again before further ball milling was continued, if necessary. Approximately 2 mg of sample was removed for analysis via PXRD (described in Section 2.2.3) at each time point required for Rietveld analysis. Around 15 mg of the sample was removed for DSC analysis (described in Section 2.2.4) after 0.5, 1, 2, 3, and 4 h of ball milling. Approximately 15 mg of the sample was removed for dynamic vapor sorption (DVS) analysis (described in Section 2.2.7) and 5 mg of the sample was removed for FTIR analysis (described in Section 2.2.8) after 4 h of ball milling.

##### 2.2.1.2 Retsch Mixer Mill MM400

LVXh was BMed using a Retsch mixer mill MM400 (Germany) at 25 Hz (1/s). A 50 mL stainless steel grinding jar was used. A sample of 2 g and three stainless steel milling balls with a diameter of 1.5 cm and a weight of 14 g each were added to the jar. This condition will be referred to as **MM400_Cond1**. Ball milling was conducted at room temperature with maximum intervals of 99 min. A 15 min break was implemented after every 99 min of milling. The samples were milled for a total duration of 4 h at time point measurements taken every minute from 1 to 10 min, and again at 0.5, 1, 2, 3, and 4 h. The samples were taken by opening the jar and removing the sample amount required for analysis using a spatula. Once the sample was removed, the jar was closed again before the continuation of further ball milling, if necessary. Around 2 mg of sample was removed for analysis via PXRD (described in Section 2.2.3) at each time point required for Rietveld analysis. Around 15 mg of sample was removed for DSC analysis (described in Section 2.2.4) at time points 0.5, 1, 2, 3, and 4 h of ball milling.

A sample of 2 g and five stainless steel milling balls with a diameter of 1.2 cm and a weight of 7 g each were added to the jar. Ball milling was conducted at room temperature with maximum intervals of 99 min. A 15 min break was implemented after every 99 min of milling. The samples were milled for a total duration of 2 h at time points taken at 3 min, 0.5, 1, and 2 h. The samples were taken by opening the jar and removing the sample amount required for analysis using a spatula. Once the sample was removed, the jar was closed again before the continuation of further ball milling, if necessary. Around 2 mg of sample was removed for analysis via PXRD (described in Section 2.2.3) at each time point for Rietveld analysis.

LVXh was BMed using a Retsch mixer mill MM400 (Germany) at 25 Hz (1/s). A 25 mL ZrO_2_ jar was used, and 0.08 g of sample was added to the jar. One ZrO_2_ ball with a diameter of 1.2 cm and a weight of 5 g was used. This condition will be referred to as **MM400_Cond2**. Ball milling was conducted at room temperature with maximum intervals of 99 min. A 15 min break was implemented after every 99 min of milling. The sample was milled for a total duration of 4 h at time point measurements taken every minute from 1 to 10 min and again at 12, 15, 20, 30, 50, 60, 70, 90, and 2, 3, and 4 h. The samples were taken by opening the jar and removing the sample amount required for analysis using a spatula. Once the sample was removed, the jar was closed again before the continuation of further ball milling, if necessary. Around 2 mg of sample was removed for analysis via PXRD (described in Section 2.2.3) at each time point for Rietveld analysis. Around 15 mg of sample was removed for analysis via DSC (described in Section 2.2.4) at time points 0.5, 1, 2, 3, and 4 h of ball milling. An additional 15 mg of sample was removed for analysis via DVS (described in Section 2.2.7) after ball milling for a total of 4 h.

#### 2.2.2 Spray Drying (SD)

A Büchi B-290 mini spray dryer (Switzerland) equipped with a nozzle fitted with a 1.5 mm cap and 0.7 mm tip was selected to spray-dried LVX. The conditions were the pump speed of 20% (7.5 mL/min) and the aspirator of 90%. Nitrogen (with a pressure of 7 bar) and air were mixed to form the desired drying gas. Water was chosen as a solvent to produce pure spray-dried (SDed) LVX. An excess of LVXh was added to 1 L of water and stirred overnight at 200 rpm using a Stuart (U.K.) hot plate stirrer SB162. The next day, the liquid was filtered under a vacuum to remove any undissolved LVX. The filtered solution was then SDed using an inlet temperature of 120 °C.

#### 2.2.3 Powder X-ray Diffraction (PXRD)

PXRD was implemented at room temperature using a benchtop Rigaku MiniflexII X-ray diffractometer (Japan) and a Haskris cooler. The samples were scanned from 5 to 40° 2θ. The settings were as follows: step width 0.05, scan rate 0.05° per second, and 1 s signal collection time per step. The tube (Cu, 1 kW normal focus) output voltage and current were operated at 30 kV and 15 mA, respectively.

#### 2.2.4 Differential Scanning Calorimetry (DSC)

DSC was executed by using a Mettler Toledo DSC (Switzerland), which was attached to an RP-100 LabPlant refrigerated cooling system (U.K.). The equipment was calibrated by an indium standard. Nitrogen was used as the purge gas. Approximately 3–5 mg samples were analyzed. Forty μL sealed aluminum pans were used, and the lids were manually pierced with three holes. Samples were heated from 25 to 250 °C at 10 °C/min.

#### 2.2.5 Modulated Differential Scanning Calorimetry (mDSC)

DSC was carried out using a Q200 Differential Scanning Calorimeter (TA Instruments, U.K.). Nitrogen was used as the purge gas (50 mL/min). Approximately 1–2 mg samples were analyzed. Sealed aluminum Hermetic pans were used, and the lids were manually pierced with three holes. Samples were equilibrated at −40 °C and modulated by ±0.80 °C every 60 s, followed by an isothermal of 2 min. Samples were ramped at 5 °C/min to 200 °C. Results were analyzed by using the TA Universal Analysis software (TA Instruments, U.K.).

#### 2.2.6 Rietveld Refinement

Rietveld refinement of the samples collected as described above was used to analyze PXRD patterns quantitatively. The method uses a set of mathematical functions to describe parametrically the PXRD patterns of pure crystalline phases, or their mixtures, based on their crystalline structure and microstructure while accounting for specimen and instrumental factors.^44,45^ For pure crystalline substances, peak intensity, width, and position provide insight into the structural and microstructural features. In the case of mixtures, the Rietveld refinement enables the quantification of the weight fraction of each crystalline phase by dividing the area under the XRD profile of each crystalline phase by the total area under the overall XRD profile. In the presence of amorphous phases, the Rietveld method can be still used, provided that the specific contribution to the diffracted intensity of the amorphous is suitably taken into account.^46^

The Rietveld analysis was performed using TOPAS 6 software. Initially, crystallographic information files (CIFs) were downloaded from the Cambridge Structural Database (CSD) and used to generate an initial function aimed at describing the experimental data. The obtained function was then matched to the experimental data by refining parameters such as the crystallite size peak intensity and background shape. Once a satisfactory match was obtained, the relative amount of crystalline and amorphous phases was estimated.

#### 2.2.7 Dynamic Vapor Sorption (DVS)

DVS was used to investigate a 4 h BMed sample of LVXh and SDed LVXam by the use of an Advantage-1 automated gravimetric vapor sorption analyzer (Surface Measurement Systems Ltd., U.K.). The temperature of analysis was set to 25.0 ± 0.1 °C. An amount of approximately 15 mg of sample was inserted into the sample carrier (stainless steel mesh) and positioned in the instrument. The sample was equilibrated at 0% relative humidity (RH), and a continual mass was attained (d*m*/d*t* ≤ 0.002 mg/min). The reference mass was documented, and the sorption (0–90% RH) and desorption (90–0% RH) programs were applied in intervals of 10% RH. At each interval, the sample mass was equilibrated (d*m*/d*t* ≤ 0.002 mg/min for approximately 10 min) or if the maximum equilibration time was exceeded before the RH was altered. The maximum equilibration times were either 5 h (method 1), 8 h (method 2), or 12 h (method 3) for the SDed sample and 5 h (method 1) for BMed powders. An isotherm was constructed from the complete sorption and desorption profile. The samples were analyzed via PXRD at the end of the sorption–desorption cycle.

#### 2.2.8 Solid-State Fourier Transform Infrared Spectroscopy (FTIR)

FTIR was conducted using a Spectrum One FTIR spectrometer (PerkinElmer) supplied with Spectrum Software version 6.1. The parameters selected were a spectral range of 450–4000 cm^–1^, a resolution of 4 cm^–1^, a scan number of 10, and a scan speed of 0.2 cm/s. KBr disks were created by direct compression using a manual hydraulic press (P&T Precision Hydraulics Ltd., Ireland). Unprocessed LVXh, BMed LVXh, and LVXam were first mixed with dried KBr using a spatula in a ratio of 1:30 for LVXh and 1:167 for LVXam. A pressure of approximately 10 bar for 1 min was applied to the sample to form the KBr discs. Deconvolution of the carbonyl region of the spectra (1800–1650 cm^–1^) was conducted to separate overlapping bands. Following subtraction of the baseline, Lorentz peak fitting was carried out on the spectra by using Origin 2018 software.

## 3 Results and Discussion

### 3.1 Ball Milling of Levofloxacin

The mechanical processing of solids by the action of ball milling relies on mechanical contact from the milling tools. The mechanical energy facilitates the collision of surfaces and unconstrained dynamic compaction. Individual powder particles experience a mix of compression and shear forces, which rely heavily on the resulting compaction rate. In turn, this depends on the mechanical action of the ball mill, such that the movement of the jar plays a major role, i.e., oscillatory or vibrational. The planetary ball mill PM100 and the mixer mill MM400 can be expected to give rise to different milling dynamics. In the case of PM100, shear/frictional processes govern the mechanochemical outcome.^47^ This tends to result in a lower rate of mechanical energy transfer to the powders and, thus, a lower milling intensity, ultimately increasing process duration. For MM400, milling dynamics is usually dominated by impact, facilitating higher milling intensities and, thus, reduced processing durations.^47,48^ Both milling methods were used and compared in this work.

#### 3.1.1 Powder X-Ray Diffraction

**Figure 2:**
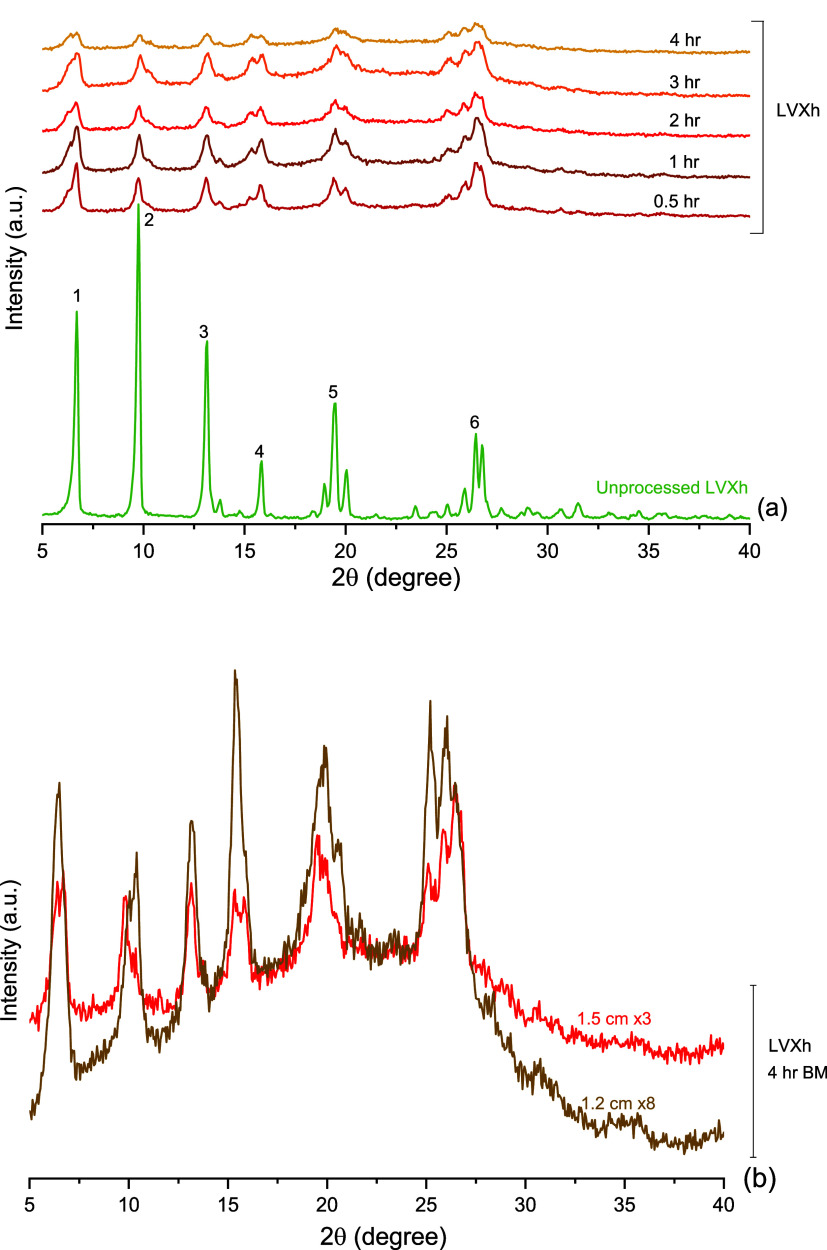
PXRD analysis of powders subjected to milling using the Retsch planetary ball mill PM100 and 50 mL stainless steel jar of (a) unprocessed LVXh and LVXh subjected to milling between 0 and 4 h using three 1.5 cm balls and (b) LVXh subjected to milling for 4 h using eight 1.2 cm and LVXh subjected to milling for 4 h using three 1.5 cm balls at 400 rpm.

Initially, the processing of LVXh by a Retsch planetary ball mill PM100 was attempted. Unprocessed LVXh depicts sharp Bragg peaks, confirming the crystalline nature of the material.^49^ The most intense LVXh is situated at 9.8° 2θ (peak 2 in Figure 2a). However, once LVXh was BMed for 0.5 h or more, the most intense peaks are visible at positions 6.7 and 26.5° 2θ (peaks 1 and 6, Figure 2). After 4 h of BMing, the peak at approximately 6.7° 2θ (peak 1) splits into two peaks. This second peak is evident throughout the ball milling process; however, it only becomes split after 4 h of BMing. Additionally, peaks marked as 2, 4, 5, and 6 (Figure 2a) undergo similar alterations to their original starting peaks when compared to those of unprocessed LVXh. The result of BMing LVXh and exposing the molecule to friction, stress, and strain could result in an alteration of the crystal structure of LVXh, visible as slight alterations of the diffractograms. Figure 2 illustrates the reduction of the Bragg peak intensity alongside peak broadening, which increased with the duration of BMing. This may indicate crystallite size reduction and the formation of crystallite lattice microstrains.^24,49^ It is evident that despite BMing for 4 h, complete abolishment of the diffraction peaks cannot be achieved.

A comparison of different ball quantities and diameters was performed. A slight reduction in intensities of Bragg peaks was observed for the powder milled using three 1.5 cm balls compared to that processed using eight 1.2 cm balls (Figure 2b). This observation may be a result of greater void volume within the jar, facilitating efficient movement of the balls. In contrast to the latter whereby the reduction of void volume may have an impact on the mixing ability, which could in turn limit the production of friction and shear forces and ultimately lead to more intense crystalline Bragg peaks.

**Figure 3:**
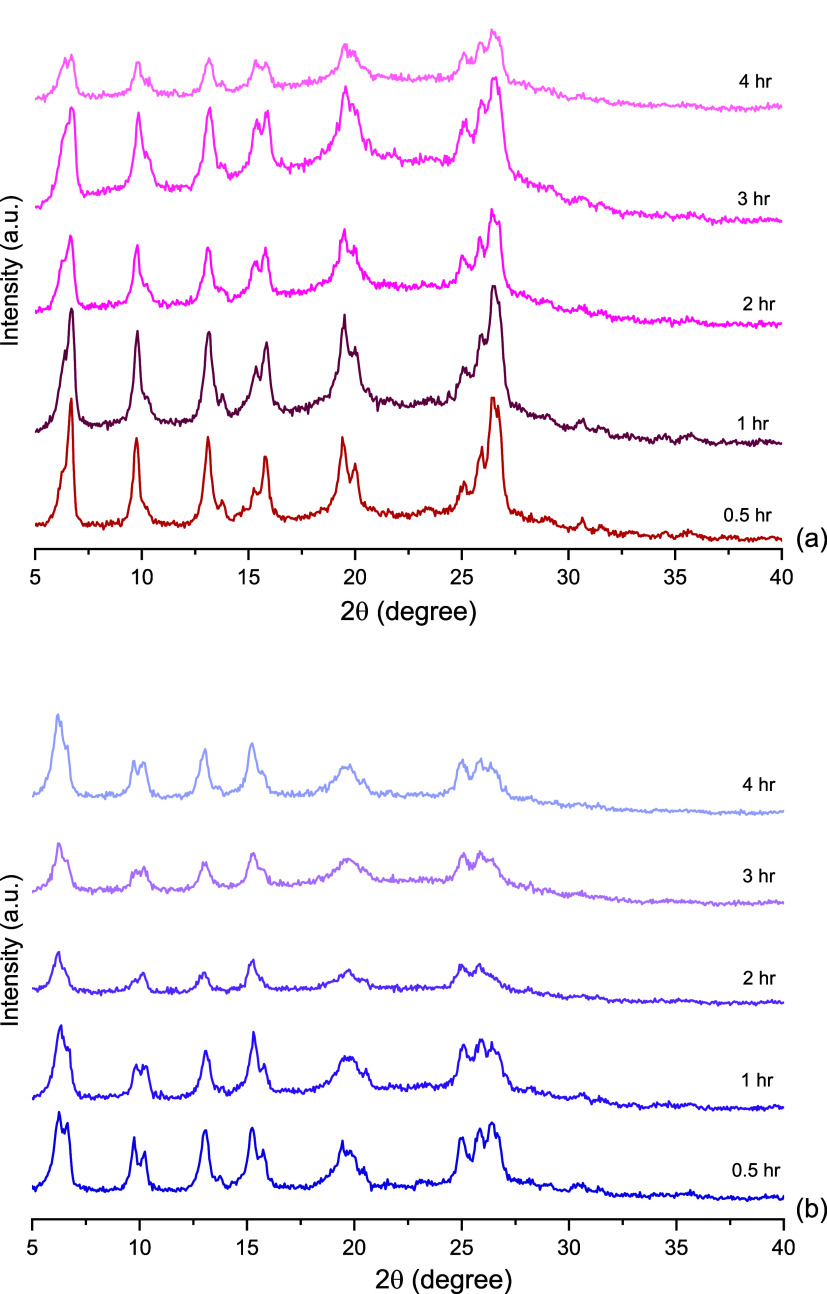
PXRD analysis of LVXh subjected to milling between 0.5 and 4 h at 25 Hz using the Retsch vibrational mixer mill MM400: (a) 50 mL stainless steel jar and three 1.5 cm balls (MM400_Cond_1) and (b) 25 mL ZrO_2_ jar and one 1.2 cm ball (MM400_Cond_2).

Subsequently, two conditions using a Retsch mixer mill MM400 were evaluated: MM400_Cond1 and MM400_Cond2 (Section 2.2.1.2). Figure 3 shows that the Bragg peaks were still present in the materials following BMing. Similarly to Figure 2, peaks 1 and 6 remain the most prominent peaks once BMed for 0.5 h or longer for MM400_Cond_1. For MM400_Cond_2, peak 1 remained the most prominent, and peak 6 slightly reduced. Variation in peak splitting is shown in both Figures 2 and 3, and slight distinctions are observable between the three conditions, i.e., PM100, MM400_Cond1, and MM400_Cond2. This may be due to the contrasting mechanical action of the two mills, which produce different primary forces.

#### 3.1.2 Thermal Analysis

**Figure 4:**
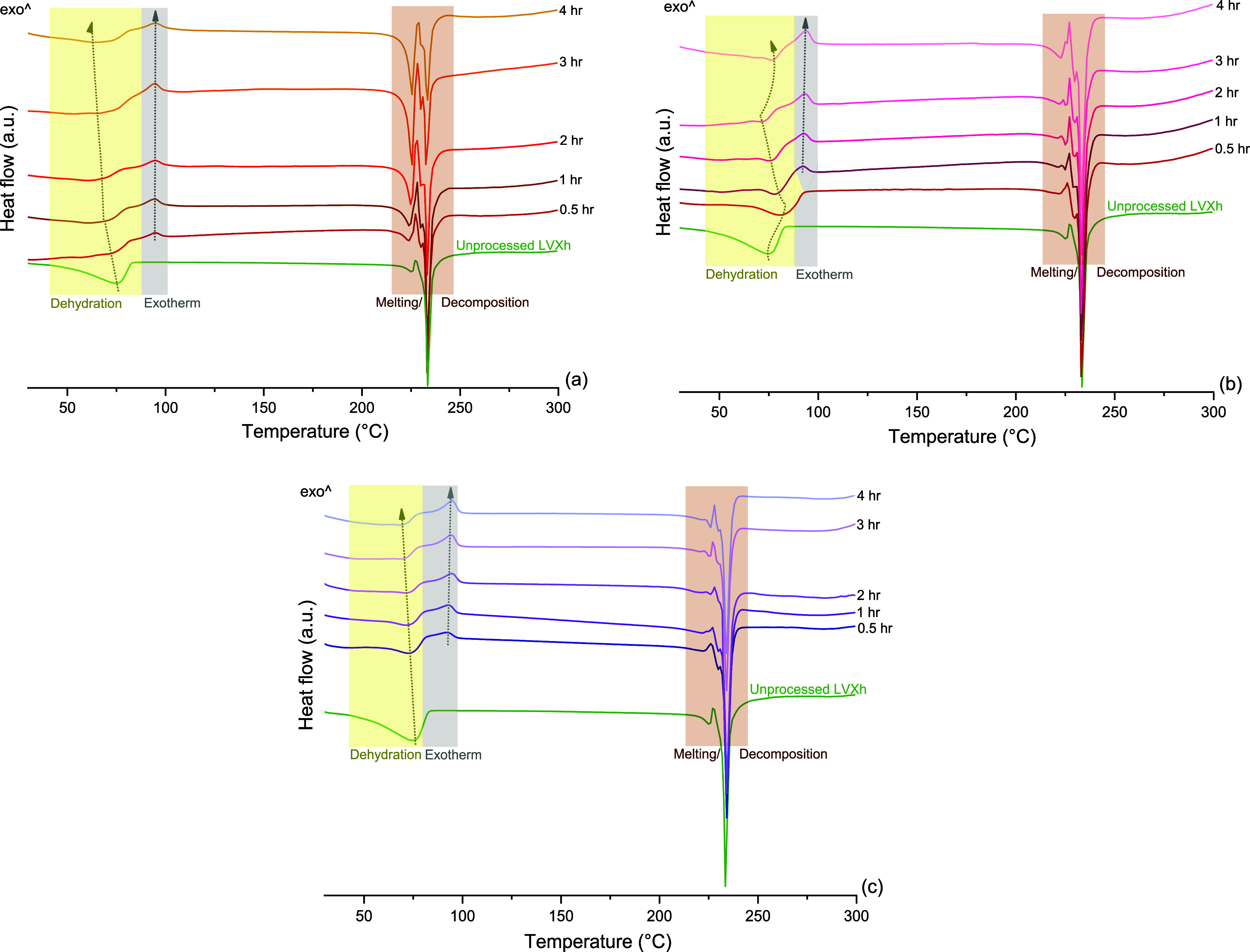
DSC analysis of unprocessed LVXh and LVXh subjected to milling between 0.5 and 4 h using (a) PM100, 50 mL stainless steel jar, and three 1.5 cm balls at 400 rpm, (b) MM400, 50 mL stainless steel jar, and three 1.5 cm balls (MM400_Cond_1), and (c) MM400, 25 mL ZrO_2_ jar, and one 1.2 cm ball (MM400_Cond_2).

Thermograms of the PM100 processed samples displayed changes with respect to the dehydration as well as the melting peaks. The DSC data for unprocessed LVXh depicts a dehydration peak at 74.8 °C (Figure 4a). When LVXh was BMed for 0.5–4 h, the dehydration peak slightly shifted to a lower temperature. After 0.5 h of BMing the peak temperature decreased to 73.5 °C, while after 4 h of milling, it was at 70.3 °C, thus continually decreasing as BMing continued. This may indicate the disruption of the crystal lattice such that the crystalline water can be liberated. This is in alignment with research, which suggests that water molecules can be released without destroying the crystal lattice by breakage of the hydrogen bonds that connect the water molecules to the piperazine rings.^39^ Thermograms of MM400 Cond_1-processed samples display divergence from not only unprocessed LVXh but also from PM100 and MM400 Cond_2 concerning the dehydration peak, which demonstrated incomplete removal of crystalline water. Interestingly, after BMing for 0.5 h, the temperature increased from 74.8 to 81.7 °C, suggesting stabilization of anhydrous/dehydrate, likely from the remaining H_2_O known to facilitate hydrogen bonding.^39^ Nevertheless, upon further processing, the temperature continually decreased; however, it remained above the starting temperature of 74.8 °C, until a processing time of 3 h, where it decreased to 72.3 °C. Despite the reduction in temperature, further processing to 4 h led to a temperature rise of 76.7 °C, surpassing yet again the starting temperature of unprocessed LVXh. The reduction and increase of the dehydration peak temperature may suggest the removal and re-entry of crystalline water, respectively. The dehydration peaks of processed samples for thermograms of MM400 Cond_2 display similarities to that of PM100 as a result of their consistent and continual decrease in peak temperature from 74.0 to 65.9 °C for 0.5 and 4 h of processing. As mentioned previously, this reduction in the temperature may indicate the release of crystalline water.

The exotherm peaks were examined for all methods upon the processing of LVXh. Unprocessed LVXh powder did not display an exotherm after the dehydration peak; however, following BMing, an exothermic peak of crystallization appeared for almost all samples, and this may indicate amorphous phase formation. It is evident that the exothermic peaks of processed PM100 samples slightly increased in temperature from 92.4 to 94.7 °C as the BMing duration increased from 0.5 to 4 h of milling, respectively. This may suggest a subtle increase in the stability of the proposed amorphous form upon further BMing. Similar to PM100 samples, MM400 Cond_1 processed samples of ≥1 h displayed an exothermic peak of crystallization as a direct result of sample processing. Comparably, the temperature increased from 92.2 to 93.8 °C for 1 and 4 h of milling. Lastly, MM400 Cond_2 depicted the appearance of an exothermic peak of crystallization for all samples. However, the exotherm remained quite consistent and only demonstrated a minute increase in temperature from 94.4 to 94.7 °C.

Unprocessed LVXh undergoes melting/decomposition at onset values of 221.6 and 231.9 °C, which were previously assigned to the melting of LVXγ and LVXβ.^39,42^ Processed LVXh by PM100, MM400 Cond_1, and MM400 Cond_2 experienced melting/decomposition between approximately 221 and 240 °C, in addition to altered thermal events unique to processed LVXh.

### 3.2 Generation of Amorphous LVX by Spray Drying

To date, fluoroquinolone drugs have never been SDed without the use of an excipient, except for CIP. CIP was previously SDed from a saturated solution made in water, which resulted in an amorphous powder. Unfortunately, CIP is practically insoluble in water; thus, the drug concentration in the solution was very low, and the process was deemed inefficient. The same group attempted spray drying of CIP from ethanol/water 9:1 (v/v), but the powder exhibited a small degree of crystallinity.^50^

**Figure 5:**
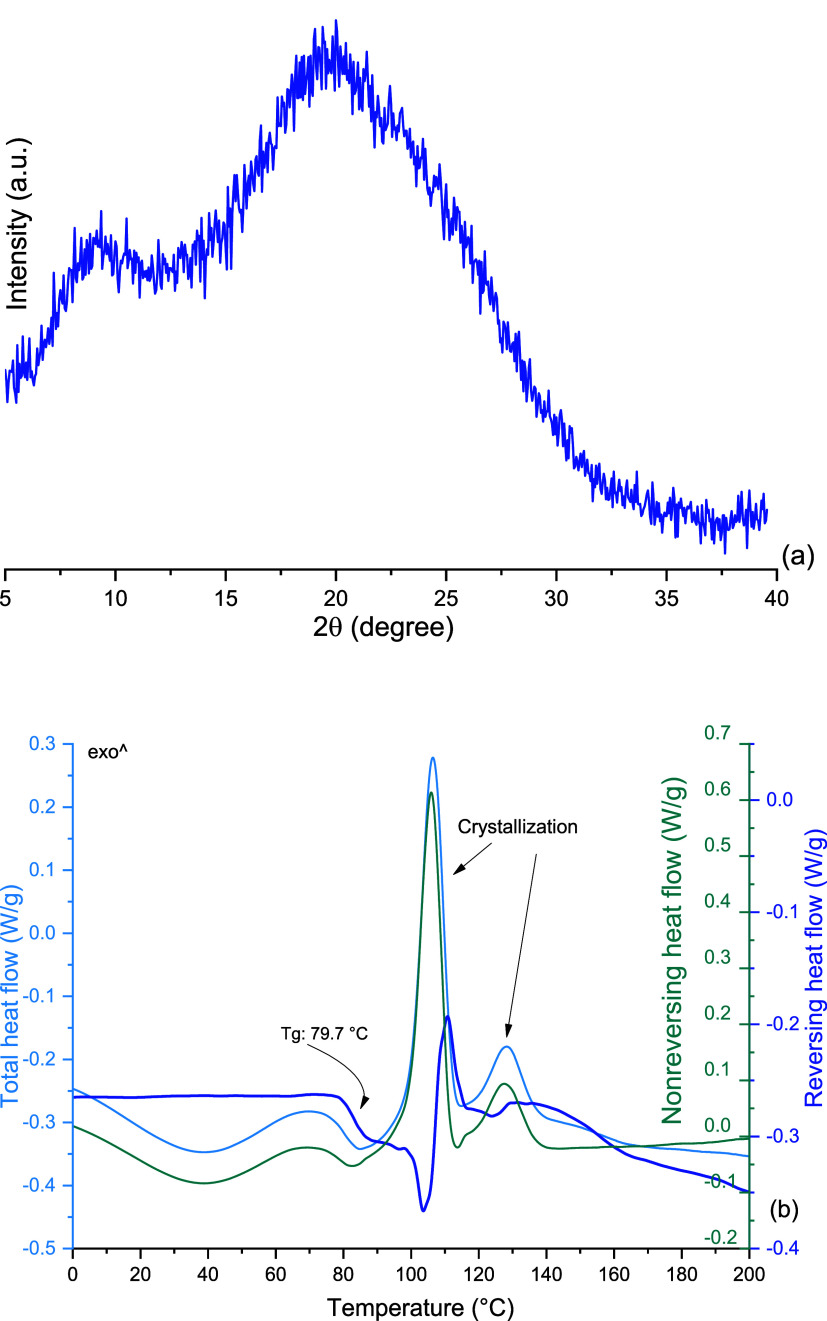
(a) PXRD analysis of spray-dried LVXam and (b) mDSC analysis of spray-dried LVXam.

LVX has never been SDed on its own to produce the amorphous form until now. Previous studies showed that LVX has been SDed to achieve complete or almost complete amorphization using metal chlorides,^51^ leucine,^52^*N*-acetylcysteine,^33^ lysozyme,^53^ and a proteolytic enzyme.^54^ LVX was SDed from a solution made in water, and this led to the formation of LVX that was completely X-ray amorphous (Figure 5a). This is portrayed by the absence of sharp Bragg peaks and, instead, the formation of a halo. It is, therefore, clear that a pure amorphous phase of LVX can be obtained using a bottom-up approach, but not the top-down, as used via milling. This behavior is similar to that of CIP, where an amorphous phase was only obtained by SDing, but not BMing.^50^

Thermal analysis of amorphous LVX (LVXam, Figure 5b) showed that its glass transition (*T*_g_) was around 80 °C and the *T*_g_ event was followed by two exothermic events, most likely of crystallization, with onset values of 102.9 and 106.2 °C. The instantaneous crystallization following the *T*_g_ represents nucleation and crystal growth and may indicate a lack of stability.^55^ This is similar to amorphous CIP, which demonstrated a crystallization peak at 121 °C, following the *T*_g_ at 86.7 °C.^50^ The *T*_g_ observed for LVXam is of a lower temperature than that of CIP, suggesting that the amorphous phase of LVX might be less stable than that of CIP.

### 3.3 Infrared Analysis

**Figure 6:**
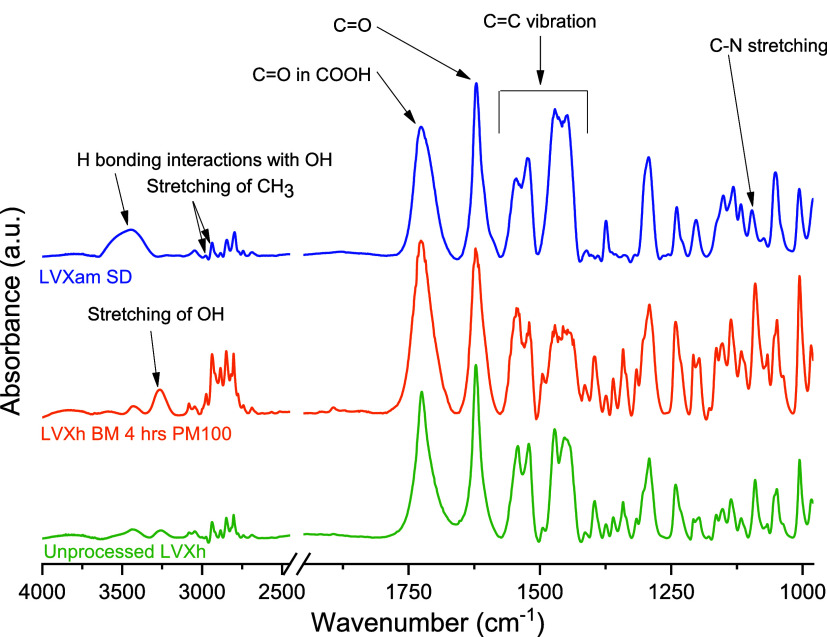
FTIR of unprocessed LVXh and LVXh subjected to milling for 4 h using PM100, and LVXam after SDing.

IR analysis was conducted for unprocessed and processed LVX (Figure 6). Unprocessed LVXh depicts an OH bonding band at 3257 cm^–1^. BMed LVXh prompts slight OH stretching at 3265 cm^–1^ and a slightly more pronounced peak.^56^ These changes could be due to H bonding interactions with OH. Moreover, LVXh SDed to produce LVXam not only engages in the stretching of OH but also participates in broadening and shifting to 3459 cm^–1^ as a likely result of H bonding interactions with OH.^57^ This broadening stretches from 3280 to 3649 cm^–1^ as opposed to the usual OH band of unprocessed LVXh stretching from 3169 to 3325 cm^–1^ and BMed LVXh stretching from 3171 to 3353 cm^–1^. Unprocessed LVXh and BMed LVXh exhibit stretching of CH_3_ at 2938 and 2975 cm^–1^,^56^ with a subtle shift to 2935 and 2978 cm^–1^ for LVXam, respectively. Unprocessed LVXh carbonyl C=O in COOH was observed at 1724 cm^–1^.^58^ BMed LVXh and LVXam carbonyl C=O in COOH were detected at 1727 cm^–1^. Unprocessed LVXh, BMed LVXh, and LVXam cyclic ketone C=O group appear at 1623 cm^–1^.^59^ The absence of peak shifting or broadening confirms that the carbonyl on the cyclic ketone C=O group is not involved in any H bonding interactions. The C=C stretching bands for unprocessed LVXh, BMed LVXh, and LVXam remain between 1413 and 1545 cm^–1^, with no signs of shifting, suggesting the absence of additional H bonding interactions. However, slight changes are depicted for SDed LVXam when compared to LVXh BMed and unprocessed LVXh whereby the disappearance of a small peak at 1492 cm^–1^ is evident, which may indicate LVXam intermolecular bonding in contrast to crystalline LVXh. Unprocessed LVXh and BMed LVXh display C–N at 1092 cm^–1^.^56^ In contrast, SDed LVXam shows the appearance of a C–N stretching at 1095 cm^–1^. In summary, the hydroxyl group OH on the carboxylic acid group underwent the most drastic shift and broadening of all reported LVXh groups and bonds. The observations made are consistent with diffractogram and thermogram findings in that the confirmation of partial or complete disruption of LVXh crystal lattice was evident.

Since slight shifts were observed for the C=O group of the carboxylic moiety, a more detailed investigation was conducted by applying a Lorentz function peak split to separate the overlapping signals of the bands. The data showed that the aggregate carbonyl stretching band is composed of two bands at approximately 1725 and 1708 cm^–1^ for unprocessed LVXh, 1727 and 1706 cm^–1^ for the BMed sample, and 1730 and 1711 cm^–1^ for the SDed sample (Figure S1), suggesting that the C=O moiety in SDed LVXam was affected by the processing. This is further confirmed by the ratio of the peak area of the band at a higher wavelength to that of the lower wavelength being 2.58, 3.11, and 0.78 for the unprocessed LVXh, BMed LVXh, and SDed LVX, respectively. This could reflect the different engagement of the carbonyl group in hydrogen bonds and/or the formation of the zwitterion form of LVX, facilitated by water, as shown in Figure 1, which was previously observed for CIP, another fluoroquinolone drug.^50^

### 3.4 Kinetic Modeling of the Mechanically Induced Phase Transformations of LVXh

**Figure 7:**
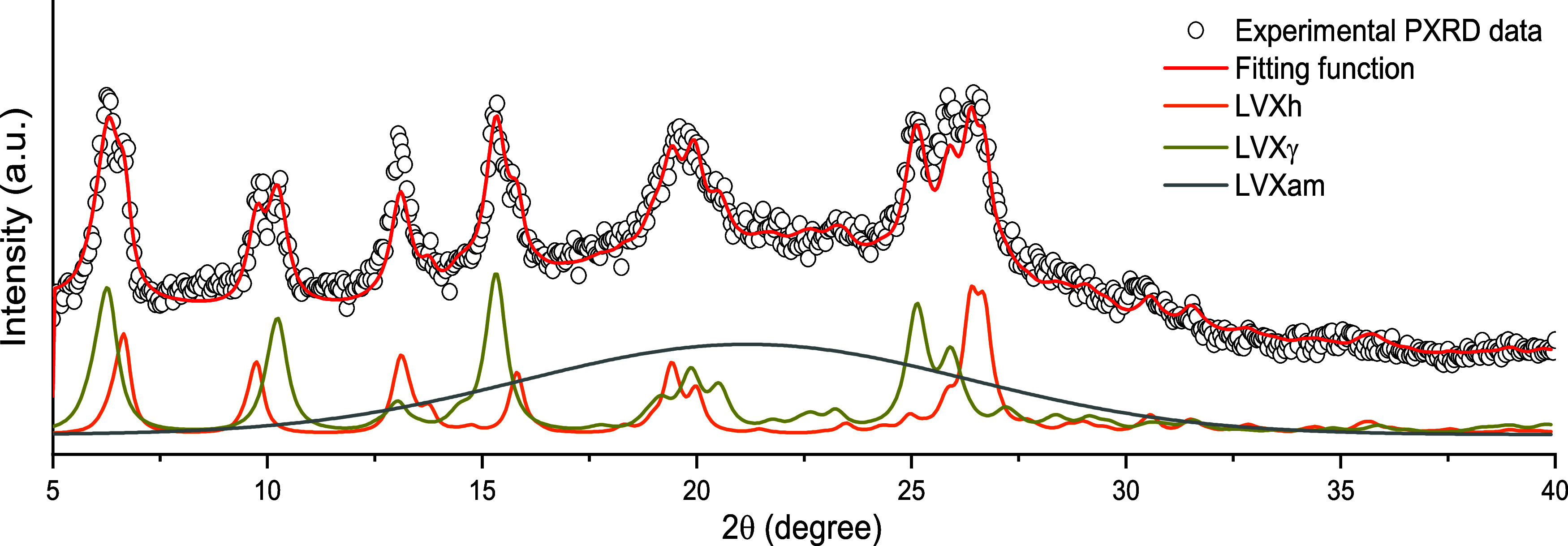
Experimental PXRD pattern and PXRD profiles obtained by the Rietveld method. Data refer to the LVXh powder ball milled for 5 min at 25 Hz using an MM400 ball mill, a 25 mL ZrO_2_ jar, and one 1.2 cm ball. The experimental points (dots) and overall Rietveld profile (red solid line), as well as the Rietveld profiles for LVXh (orange solid line), LVXγ (maple solid line), and LVXam (gray solid line) are shown.

The Rietveld method was used to analyze the different sets of the PXRD data obtained from LVXh processed using MM400 and PM100 (Figures SI.2–SI.4). An example of this approach is presented in Figure 7, and it is evident that the experimental data points and Rietveld fit profiles incomparably overlap. This signifies an accurate estimate of the relative quantity of different phases, which exist upon mechanical processing of LVXh, i.e., LVXh, LVXhγ, and LVXam.

The quantitative PXRD analysis reveals that irrespective of the mechanical processing conditions, ball milling induces, first, the formation of the γ phase and then the appearance of an amorphous phase. Although the identified LVXγ and LVXam phases are mixtures, a new method for forming these polymorphs from LVXh has been established. Previous studies described the formation of LVXγ by dehydration of LVXh above 70 °C under nitrogen conditions.^39,42^ Further investigations succeeded in producing pure LVXγ from LVXh at 150 °C in synthetic air.^40^ Multiple LVXh solvates were found to convert to LVXγ by desolvation, i.e., heating and solvent removal.^41^

Although multiple water-free phases exist for LVX (Figure 1), they vary in the conformation orientation of the piperazine ring, which influences the ease and/or mechanism of dehydrate rehydration, making some water-free forms slightly more or less unstable than others.^13^ LVXγ, although unstable at ambient conditions, may be the more stable anhydrous/dehydrate due to the presence of unchanneled voids surrounded by rigid methyl groups, which, in combination, prevent water from reentering without the complete collapse of the crystal structure first.^40^ This is in contrast to water-free LVXα, which can directly rehydrate without the collapse of the crystal structure. Additionally, the pure form of LVXα has yet to be formed from the dehydration of LVXh^41^ (Figure 1b), making it a less likely candidate to form under milling conditions. The necessity for LVXγ to collapse before rehydrating may explain the transition from LVXγ to LVXam. According to the literature, it is also possible for LVXγ to convert to LVXδ if cooled below 54 °C while remaining absent of any moisture.^40^ LVXδ did not transpire in this study as the reaction proceeded in a closed vessel and was only opened for sample removal. The likelihood of the escape of any free water molecules was thus limited. In any case, the reentrance of moisture would be possible upon brief exposure to atmospheric conditions if water were to escape, confirming that moisture-free conditions were not provided.

To interpret the kinetic evidence and gain deeper insight into the observed transformations, a simple kinetic model was implemented. The model was based on several assumptions, which considered, at least to a first approximation, the statistical nature of the mechanical processing by ball milling and the stress conditions experienced by the powder during individual impacts. It was assumed that, during individual impacts, the mechanical loading exceeds a certain threshold in a set of small subvolumes v* that are located in the volume of compressed powder. The total volume of powder affected by critical loading conditions (CLCs), *v*, during individual impacts is the sum of the volumes v*. If the powder inside the jar is effectively stirred and the volumes v* are stochastically involved in any given impact, the statistics of v* affected by CLCs can be described analytically with κ being the ratio between *v* and the total volume of powder, *V*, inside the jar. The volume fraction of powder, χ_*i*_(*m*), that has undergone CLCs *i* times after *m* impacts can be expressed as eq 1
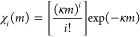
1Equation 1 represents the probability that CLCs affect a total volume *v* under the hypothesis that impacts always involve the same fraction of powder, κ. Although it describes how volumes v* undergo CLCs, it does not consider the possible changes induced by CLCs in volumes v*. In this regard, it is reasonable to expect that specific values α_*i*_ of the degree of transformation in volumes v* after *i* CLCs can be associated with volume fractions χ_*i*_(*m*). Accordingly, the total degree of transformation, α(*m*), can be written as eq 2
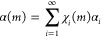
2which can be regarded as a weighted average of the transformation degree over the different volume fractions χ_*i*_(*m*).

Equation 2 relates to the volume *v* effectively involved in CLCs to the degree of transformation. In the absence of more detailed information, it is reasonable to keep the assumptions that allow obtaining transformation kinetics in line with experimental evidence as simple as possible. Thus, it can be noted that eq 2 results in an exponential decrease of the initial LVXh phase if it is hypothesized that the degree of transformation from LVXh to LVXγ in individual volumes v* changes abruptly from 0 to 1 as the volumes undergo a single CLC. In other words, this hypothesis implies that the entire volume *v* is involved in the formation of LVXγ after the first CLC. In such volume, the transformation is complete. Following on from the first impact, the powder inside the jar comprises a volume fraction of LVXh, χ_LVXh_(1), equal to 1 – κ_1_, and a volume fraction of intermediate LVXγ χ_γ_(1), equal to κ_1_. Upon the second impact, CLCs can involve both the residual LVXh phase and the newly formed LVXγ. It may be expected that LVXh keeps transforming into LVXγ with a rate constant equal to that of κ_1_. However, it is reasonable to hypothesize that the intermediate LVXγ can, on the one hand, partially reverse the transformation and form LVXh with a rate constant equal to κ_–1_ and, on the other, give rise to the amorphous phase with a rate constant equal to κ_2_. Accordingly, after the second impact, the volume fractions of LVXh, χ_LVXh_(2), LVXγ anhydrous phase, χ_γ_(2), and amorphous phase, χ_am_(2), are equal to 1 – κ_1_ – κ_1_(1 – κ_1_) + κ_1_ κ_–1_, κ_1_ + κ_1_(1 – κ_1_) – κ_1_ κ_–1_ – κ_1_ κ_2_, and κ_1_ κ_2_, respectively. With the assumption that the intermediate LVXγ and amorphous phases can partially reverse their transformation, it is possible to write the following kinetic scheme:
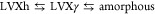
Since the observed transformations occur in relatively long-time intervals, this denotes that the rate constants are much smaller than 1. Thus, the following kinetic equations can be defined (eqs 3–5):
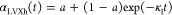
3
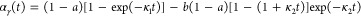
4
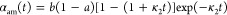
5where κ_1_ and κ_2_ can be regarded as rate constants, while *a* and *b* account for the coexistence of three solid phases. Specifically, *a* represents the fraction of LVXh attained at the end of the transformation, and *b*(1 – *a*) represents the final fraction of the amorphous phase.^60^

**Figure 8:**
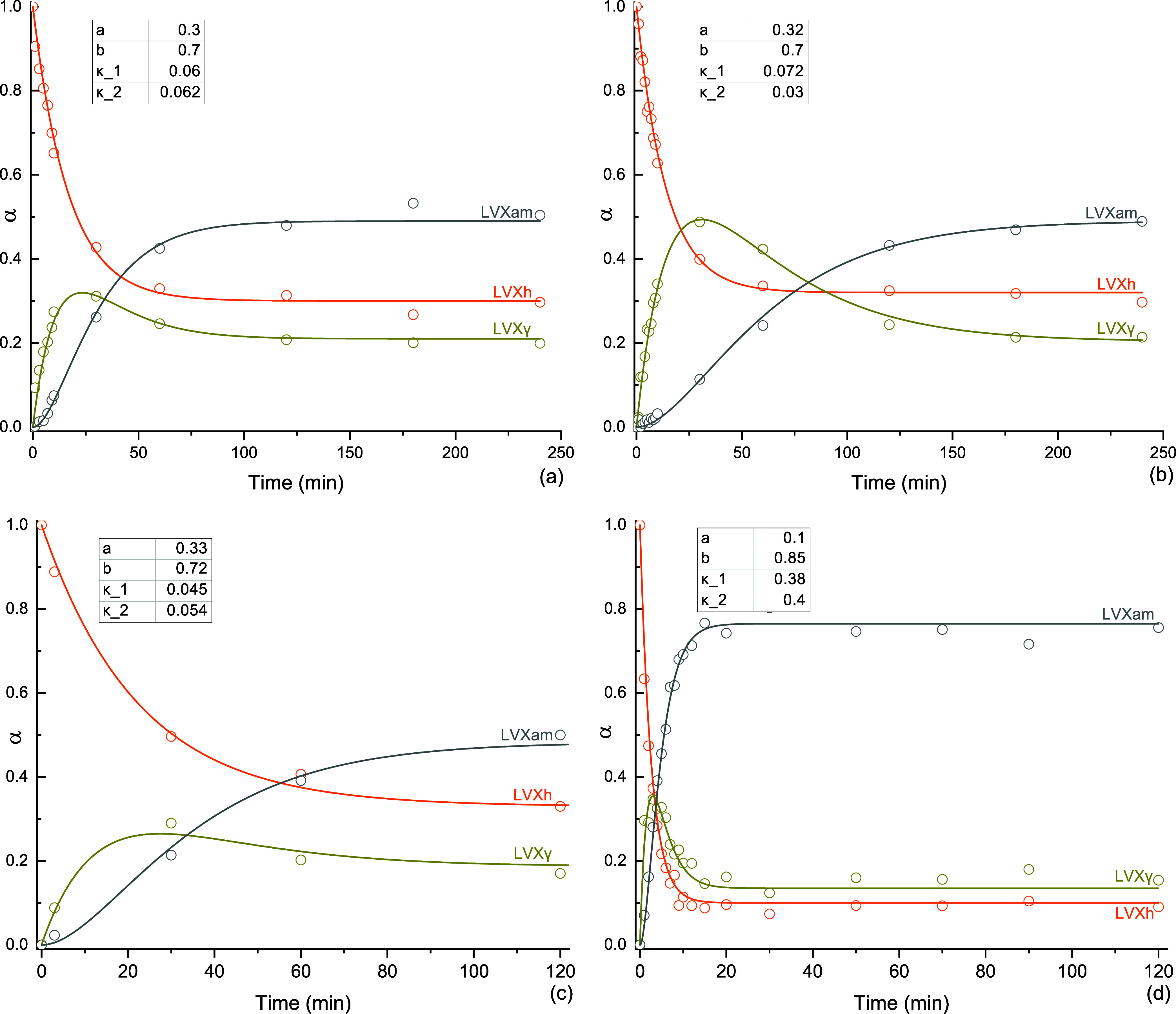
Fraction (α) of LVXh (orange), LVXγ (maple), and LVXam (gray) as a function of milling time, *t*. Experiments carried out with (a) PM100 ball mill and 50 mL stainless steel jar using three 1.5 cm balls at 400 rpm, (b) MM400 ball mill operated at 25 Hz with the 50 mL stainless steel jar and three 1.5 cm balls, MM400_Cond1, (c) MM400 ball mill operated at 25 Hz with the 50 mL stainless steel jar and five 1.2 cm balls, and (d) MM400 ball mill and 25 mL ZrO_2_ jar using one 1.2 cm ball at 25 Hz—MM400_Cond2. Fitting parameters a, b, κ_1_, and κ_2_ are included. The unit for κ_1_ and κ_2_ is min^–1^.

Equations 3–5 can be numerically solved and best fitted to experimental data. The obtained kinetic curves are shown in Figure 8. It was depicted that the phase transformation indeed has a consecutive character, so LVXγ must be regarded as an intermediate between the starting phase, which is LVXh, and the final amorphous material (LVXam).

Using PM100, LVXγ achieved α maximum of 0.32 at approximately 25 min of milling before reducing to α = 0.21 at the end of milling. The amount of LVXam at the end of the process (2 h) was around 50%. The quantities of LVXγ and LVXam remained constant at a steady state at approximately 105 min of milling (Figure 8a). For MM400_Cond1, the LVXγ phase achieved α maximum of 0.49 at approximately 25 min of milling before reducing to α = 0.21. Similar to PM100, LVXam obtained α maximum = 0.49 (Figure 8b). Increasing the number of balls from three to five did not affect the quantities of LVXγ and LVXam considerably (Figure 8c), and the steady state was achieved after around 100 min. For MM400 Cond_2, the LVXγ phase achieved α maximum = 0.35 at approximately 3 min of milling before reducing to α = 0.14. These conditions gave the greatest amount of LVXam, α = 0.77. The levels of LVXγ and LVXam remained constant upon reaching steady state at approximately 20 min of milling (Figure 8d).

To summarize, maximum quantities of LVXγ phase were reached for PM100 and MM400 Cond_1 after 25 min of milling and for MM400 Cond_2 after 3 min of milling. Additionally, around 50% conversion to LVXam was achieved using PM100 and MM400 Cond_1 compared to MM400 Cond_2 where around 80% was amorphized. The degree of the disorder appears to be in agreement with the PXRD data presented in Section 3.1.1 (Figure 3). The best-fitted values and final fractions of LVXh, LVXγ, and LVXam for PM100 and MM400_Cond1 are very similar, while slight differences in the κ_1_ and κ_2_ values are observed (Figure 8). This suggests that the mechanochemical transformation was relatively insensitive to changes in the mechanical action of the mill or the frequency of impacts. This contrasts with the final fractions and rate of transformation of LVXh, LVXγ, and LVXam for MM400_Cond2, whereby the rate of transformation was remarkably faster. However, it should be noted that a smaller amount of powder, 0.1 g, was used in experiments with the ZrO_2_ jar, while 2 g was used when a stainless steel jar was employed.

The ability to quantify the polymorphs of APIs is extremely important for optimal polymorph selection for the formulation. Methods, such as IR, Raman, and solid-state nuclear magnetic resonance (SSNMR), are commonly used as a quantitative method for polymorphs,^61,62^ including amorphous forms.^63^ However, in more recent years, Rietveld refinement has been used for this purpose in isolation or in combination with more traditional methods described due to its accurate analysis^61^ and efficient procedure. Rietveld refinement was utilized as a quantitative method for analyzing the stability, transformations, kinetics, and crystal domain size alterations of suspensions of mebendazole in the solid state.^61^ Venlafaxine hydrochloride^62^ and carbamazepine^64,65^ were also investigated for quantification, identification,^62,64,65^ and purity analysis of polymorphs detected.^62^ In addition, this approach has also been used to quantify the crystallinity of recrystallized amorphous solid dispersions of fenofibrate and ketoconazole.^63^ The accuracy of the results obtained the ability to detect and quantify the different forms simultaneously using conventional PXRD data, and the speed of analysis make Rietveld refinement an advantageous quantification method for phase transformation in the solid-state, as shown in this work.

### 3.5 Physical Stability of Processed LVX

**Figure 9:**
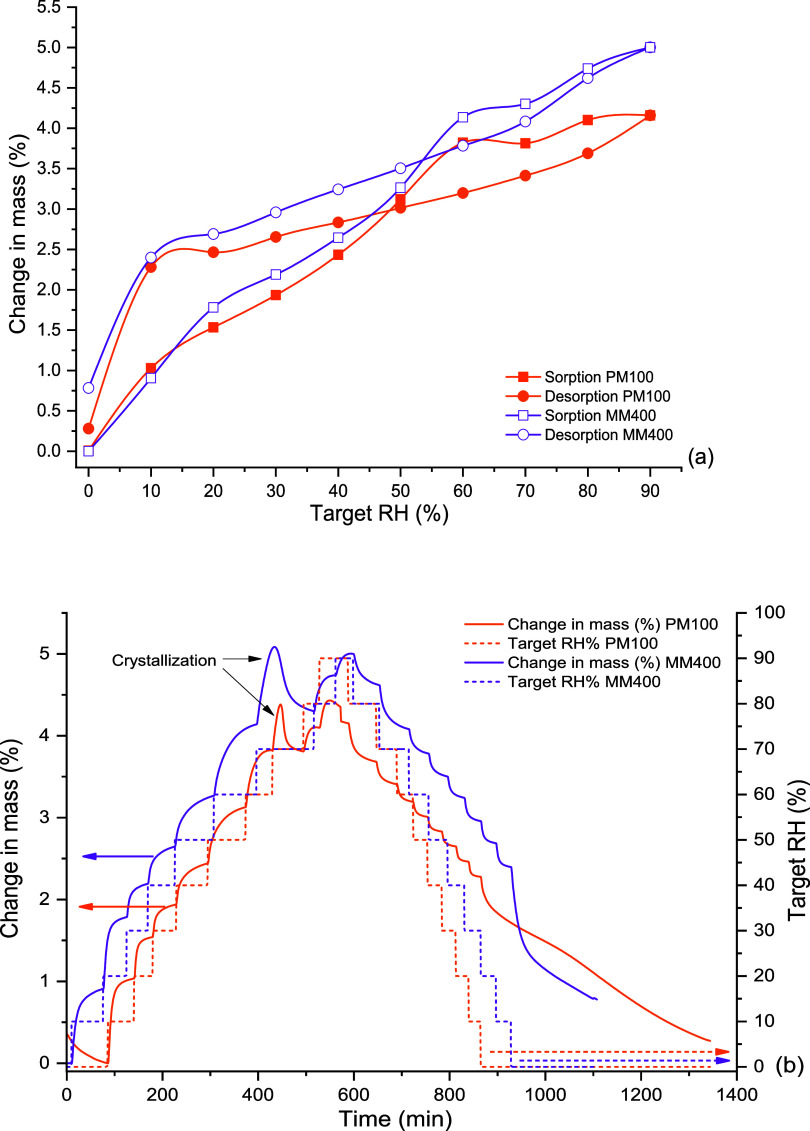
DVS plots of LVXh BMed for 4 h: (a) isotherm plots of LVXh sample BMed at 400 rpm using PM100 and BMed at 25 Hz using MM400_Cond_2 and (b) mass plot over time of LVXh sample BMed at 400 rpm using PM100 and BMed at 25 Hz using MM400_Cond_2.

DVS was used to analyze LVXh after 4 h of ball milling using PM100, MM400 Cond_2, and SDed LVXam. These analyses were carried out to evaluate the effect of moisture on the solid-state stability of the BMed sample in comparison to that of the SDed sample. Figure 9 depicts a sample mass increase when the relative humidity (RH) increases for BMed LVXh. There was evidence of partial amorphization and/or crystal lattice defects as the LVXh sample milled for 4 h crystallized at 70% RH during the analysis and overall, and the powder sorbed around 4% w/w of moisture at 90% RH. In contrast, the desorption plot illustrates a rapid decrease in mass (%), which highlights the quick weight loss and dehydration of the sample. The detection of amorphization is in agreement with the exothermic crystallization peak obtained for BMed samples (Figure 4) and the amorphization confirmation utilizing Rietveld analysis (Figure 7). The sample BMed by PM100 lost approximately 2% w/w moisture between 90 and 10% RH (desorption) over 300 min, followed by another loss of 2% w/w moisture between 10 and 0% RH over 450 min. The sample in BMed by MM400 lost approximately 2.5% w/w moisture between 90 and 10% RH over 300 min and subsequently by a further loss of approximately 1.5% w/w moisture between 10 and 0% RH over 200 min.

**Figure 10:**
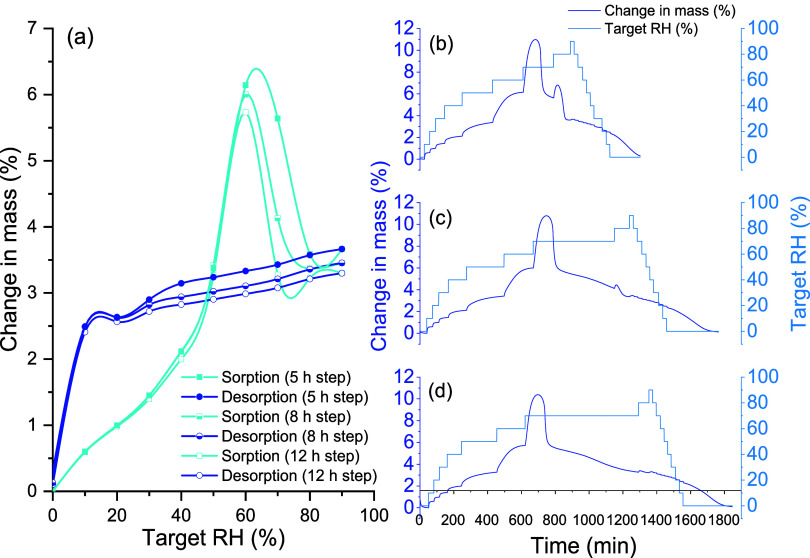
DVS plots of SDed LVXam: (a) isotherm plot using method 1 (5 h step), 2 (8 h step), and 3 (12 h step), (b) mass plot over time using method 1, (c) mass plot over time using method 2, and (d) mass plot over time using method 3.

The LVXam powder obtained by SDing sorbed more moisture than the semicrystalline BMed LVXh powder with sorption reaching ≥5% w/w of moisture at around 60–70% RH for methods 1, 2, and 3 (Figure 10). The ability to sorb large amounts of moisture is in alignment with the characteristics of amorphous materials, which are known to sorb more moisture compared to crystalline or semicrystalline materials. The sample subjected to DVS methods 1 and 2 lost 1% moisture between 90 and 10% RH (desorption) over 200 min and method 3 over 150 min. The moisture loss is comparable for all methods; however, the sample analyzed using method 3 differs in the duration of moisture loss. The mass/kinetic plots for samples ran using methods 1 and 2 show that the majority of the sample crystallized at 70% RH, with residual amounts crystallizing at 80% RH. Method 3 facilitated a longer drying cycle of 12 h, which allowed the sample to completely crystallize after 3 h of exposure at 70% RH.

Thus, both amorphous phases generated by BMing and SDing crystallize at the same % RH (Figures 9 and 10), revealing their similar capabilities to withstand crystallization under moisture conditions. Additionally, the crystallization peaks obtained for the SDed sample (Figure 10) are substantially larger compared to those shown for the BMed samples (Figure 9). This is as expected since the starting material of the SDed sample was completely amorphous (Figure 5), in contrast to the BMed sample, which was semicrystalline in nature (Figures 2–4). DVS and post-DVS PXRD analyses showed that all processed samples (BMed and SDed) crystallized to the most stable hemihydrate form, matching the 2.5% water weight of LVXh.

## 4 Conclusions

Solid-state transformations of levofloxacin hemihydrate (LVXh) were examined primarily in the milling process using spray drying as a comparative method. The solid-state changes were detected using PXRD, DSC, FTIR, Rietveld, and DVS analyses. Different mechanical actions and parameters demonstrated crystallinity reduction of the diffractograms of LVXh upon processing using PM100 and MM400, with MM400 demonstrating the greatest Bragg peak reductions for both methods, i.e., MM400 Cond_1 and MM400 Cond_2. Thermograms of PM100 and MM400 Cond_2 suggested disruption of the crystal lattice, facilitating the liberation of the crystalline water. The thermograms of the samples subjected to milling revealed an exothermic peak of crystallization. The presence of an anhydrous/dehydrated form and the event of crystallization were related to LVXγ and LVXam, respectively, by the Rietveld method. The mechanism discovered established that LVXh first transforms to the anhydrous/dehydrated LVX form γ (LVXγ) as an intermediate phase, before transpiring to LVXam. This is particularly interesting since LVXam was previously reported to form directly from LVXh upon dehydration. Additionally, LVXγ was only ever obtained by dehydration/heating and desolvation methods, according to the literature. Thus, a new method of forming polymorphs by the process of milling was developed, and the mechanism by which LVXγ plays an essential role in achieving amorphization was revealed. Rietveld method revealed that PM100 and MM400 Cond_1 had longer rates of transformation of LVXγ and contained more LVXγ form compared to MM400 Cond_2. The use of MM400 Cond_2 resulted in approximately 80% conversion of LVXh to LVXam compared with other methods. Overall, differences in the final fractions, κ_1_ and κ_2_ values, and rate of transformations were obtained. Despite incomplete amorphization upon utilization of the milling process, complete amorphization of LVX was achieved by spray drying from water. The comparison of BMing and SDing revealed that the outcome of amorphization is both mechanism- and process-dependent. The pure phase of LVXam was reported for the first time in the literature as having a glass transition of around 80 °C. DVS depicted that both amorphous phases generated by BMing and SDing crystallize at 70% RH, revealing their similar capabilities to withstand crystallization under moisture conditions. The samples crystallized to the most stable hemihydrate form upon analysis. These findings are valuable as the new discoveries obtained may be transferred and/or applied to current and future fluoroquinolone drugs with similar molecular structures. In addition, these discoveries contribute toward a greater understanding of mechano/tribochemistry and, in turn, can help to fill the gaps of solid-state mechanochemistry as the future of pharmaceutics moves toward a greener world.

## Supporting Information Available

FTIR peak deconvolution for (a) LVXh as received, and (b) ball-milled LVXh and spray-dried LVX (Figure S1); experimental PXRD patterns of LVXh subjected to ball milling using the Retsch planetary ball mill PM100 and 50 mL stainless steel jar (Figure S2); experimental PXRD patterns of LVXh subjected to ball milling using the MM400 mill working at 25 Hz (Figure S3); and experimental PXRD patterns of LVXh subjected to ball milling using the MM400 mill at 25 Hz (Figure S4) (PDF)
