# Supplementary material for: Mechanochemically Induced Solid-State Transformations of Levofloxacin
Source: Mol Pharm. 2024 Apr 25;21(6):2838–53. doi: 10.1021/acs.molpharmaceut.4c00008 (PMC11151213; doi:10.1021/acs.molpharmaceut.4c00008)
Supplement: Supplementary file 1 — mp4c00008_si_001.pdf [file mp4c00008_si_001.pdf]

Supporting Information file

Mechanochemically induced solid-state transformations of levofloxacin

Lena Kadri, Maria Carta, Giulio Lampronti, Francesco Delogu, Lidia Tajber

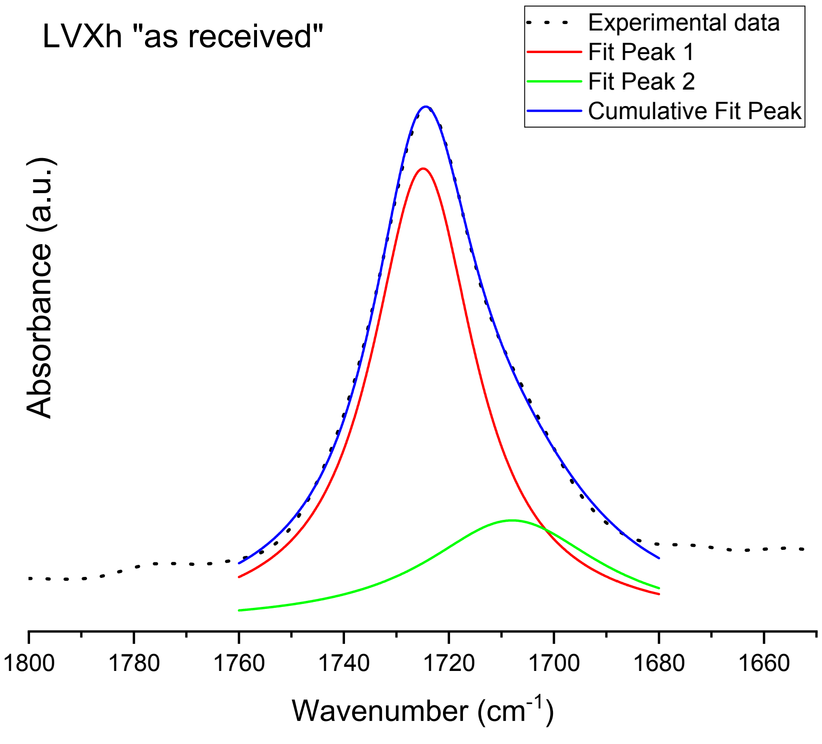

a)

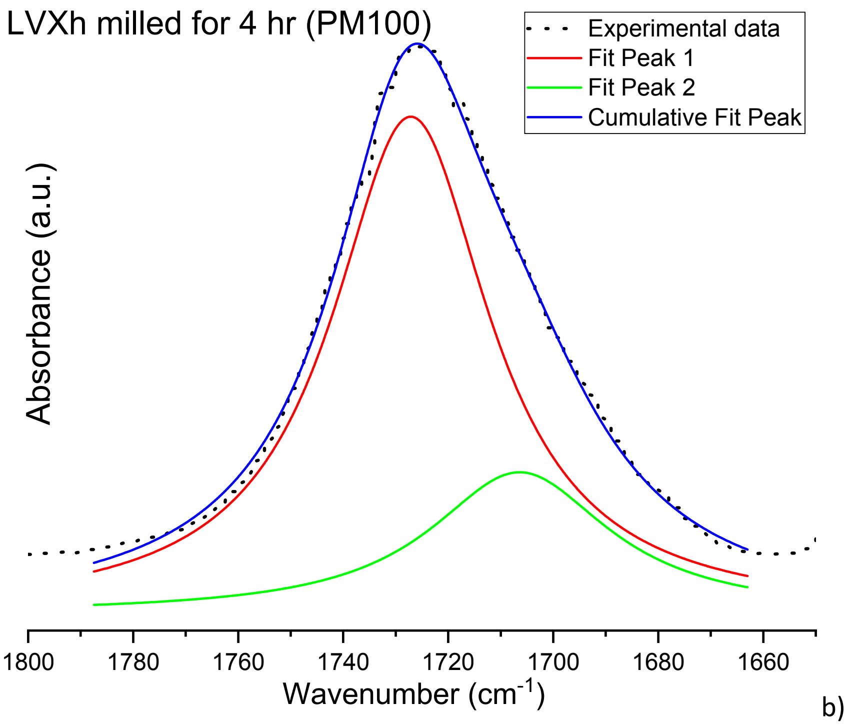

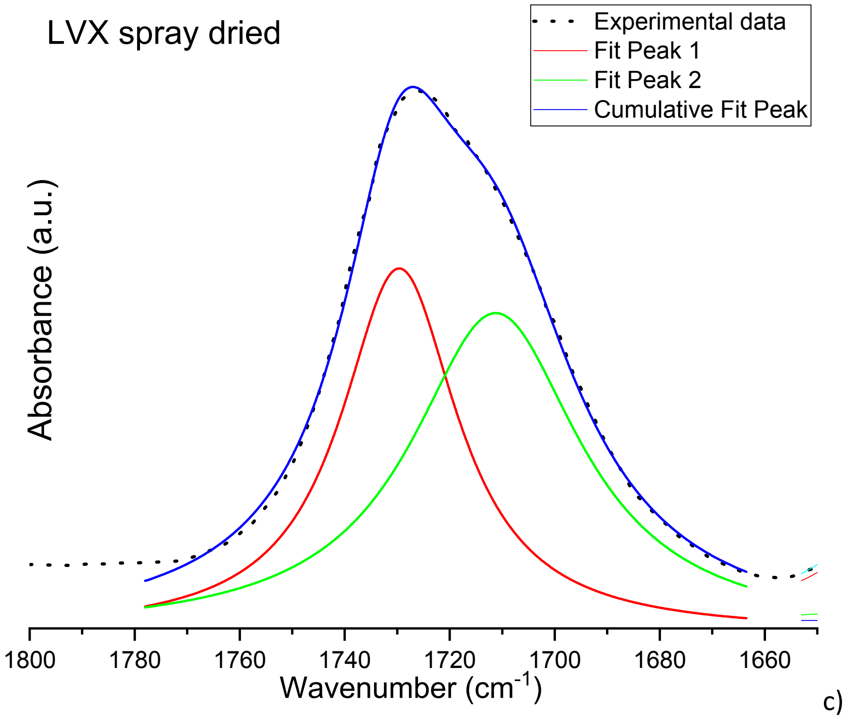

Figure S1. FTIR peak deconvolution for a) LVXh as received, b) ball milled LVXh and spray dried LVX.

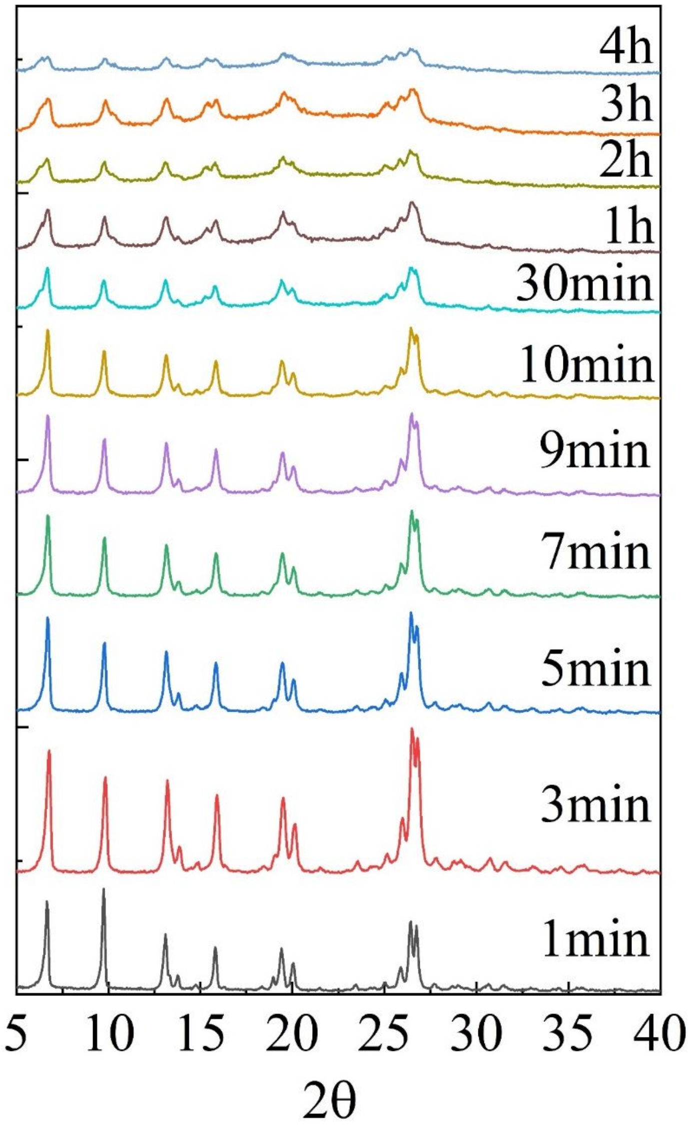

Figure S2. Experimental PXRD patterns of LVXh subjected to ball milling using the Retsch planetary ball mill PM100 and 50 mL stainless steel jar. Data refer to different processing times, ranging from 1 min of ball milling to 4 hours.

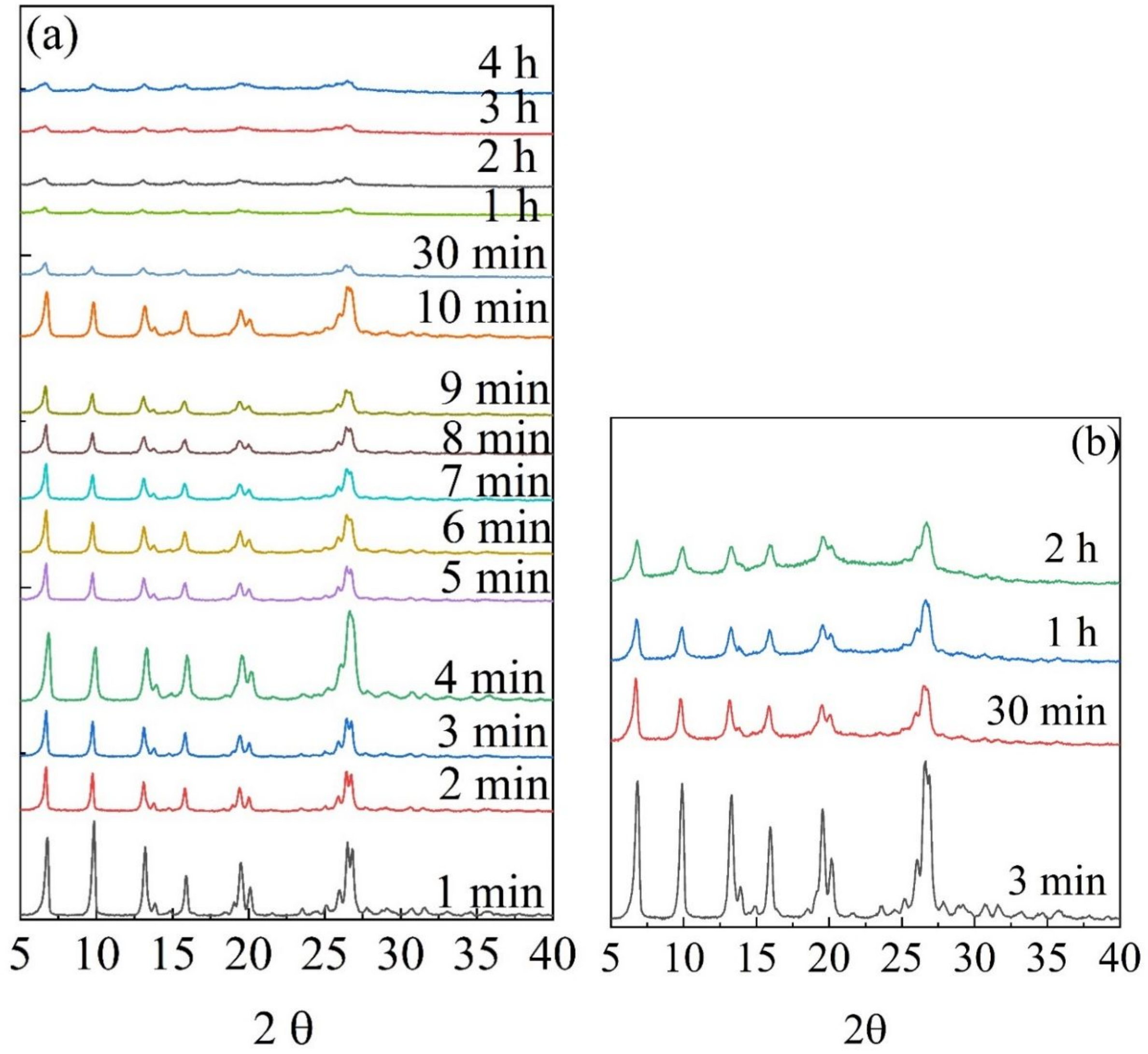

Figure S3. Experimental PXRD patterns of LVXh subjected to ball milling using the MM400 mill working at 25 Hz. The mill was equipped with a 50 mL stainless steel jar and (a) 3 stainless steel balls, (b) 5 stainless steel balls. Data refer to different processing times, ranging from 1 minute of ball milling to 4 hours.

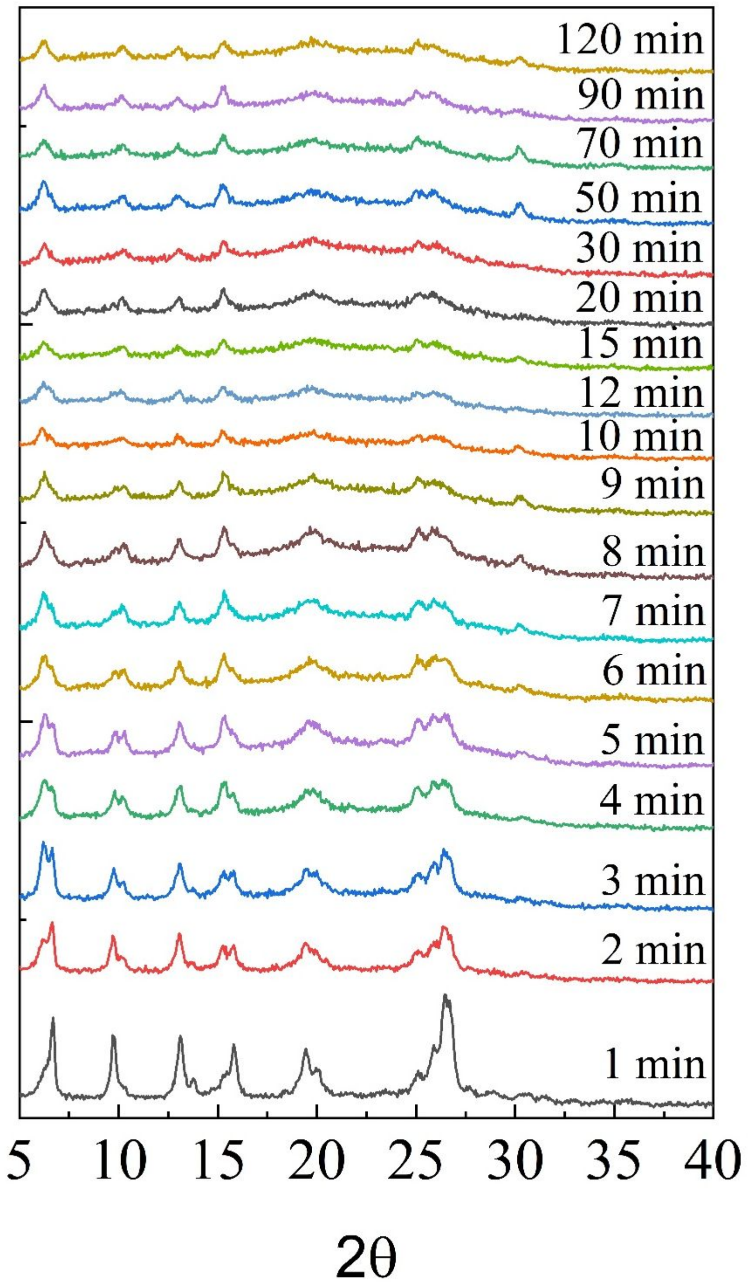

Figure S4. Experimental PXRD patterns of LVXh subjected to ball milling using the MM400 mill at 25 Hz. The mill was equipped with a 25 mL zirconium jar and one zirconium ball. Data refer to different processing times, ranging from 1 min of ball milling to 2 hours.
